# Primary plasma cell leukemia with light chain secretion and multiple chromosomal abnormalities: How successfully treated? – A case report with review of literature

**DOI:** 10.4103/0971-5851.73603

**Published:** 2010

**Authors:** Manu Goyal, Noorjahan Mohammad, Satya Dattatreya Palanki, Salil N. Vaniawala

**Affiliations:** *Department of Laboratory Medicine, Indo-American Cancer Institute and Research Centre, Hyderabad, India*; 1 *Department of Medical Oncology, Indo-American Cancer Institute and Research Centre, Hyderabad, India*; 2 *Cytogenetics and Molecular Biology, Gene Lab, Nanpura, Surat - 395 001, India*

**Keywords:** *Flow cytometry*, *fluorescent in-situ hybridization*, *free light chains*, *primary plasma cell leukemia*, *transplant*

## Abstract

Primary plasma cell leukemia is a rare form of plasma cell dyscrasia. We present a case which had leukocytosis with numerous circulating plasma cells in the peripheral blood. Flow cytometry revealed an unusual CD117 expression. Free light chain analysis in the serum showed a markedly elevated level of free lambda light chains. Radiography did not reveal any lytic lesions. Fluorescent in-situ hybridization analysis revealed deletion of 13q14.3 and t(4;14)/t(11;14), while the cytogenetic analysis was normal. The patient was given chemotherapy and was subjected to autologous stem cell transplant, after which she is in complete remission till date.

## INTRODUCTION

Plasma cell leukemia (PCL) is a rare form of leukemia and occurs in 1–2% of patients with multiple myeloma (MM).[1–4] These are characterized by presence of more than 20% plasma cells in the peripheral blood and an absolute plasma cell count of more than 2×10^9^/L.[4–6] There are two forms of PCL: primary and secondary.[1–3,5,7] The primary form occurs in the individuals without a preceding MM, whereas the secondary form is a leukemic transformation in individuals with MM.[1–3,5,7] Primary PCL (PPCL) has been estimated to occur in less than one case in a million. We report a case of PPCL occurring in a 40-year-old female, with light chain secretion, flow cytometry revealing an unusual CD117 expression and multiple karyotypic abnormalities detected on fluorescent *in-situ* hybridization (FISH).

## CASE REPORT

**Figure 1:**
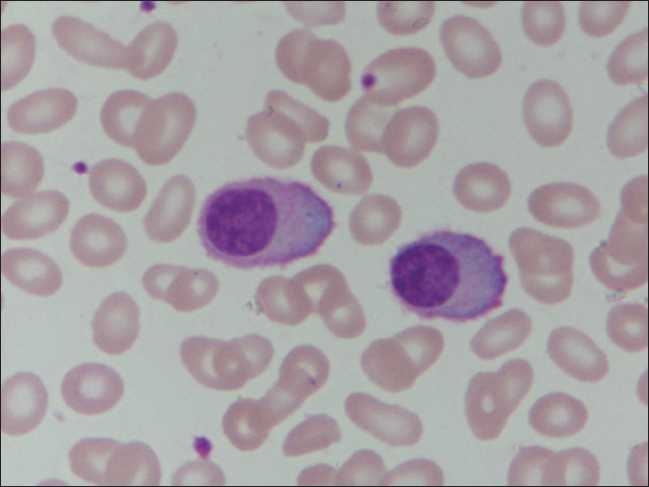
Peripheral smear showing marked prominence of plasma cells having abundant basophilic cytoplasm and eccentrically placed nucleus with clumped chromatin (Leishman, ×1000)

A 40-year-old female had presented with conjunctival hemorrhage. She had giddiness, fatigue, loss of appetite and weight; there was no fever or organomegaly. She had erythematous skin rash over the upper trunk, associated with itching, which was present for a year and was not bothersome. Her peripheral blood counts revealed the following: hemoglobin 10.6 g/dL, total leukocyte count 39.6×10^9^/L and platelet count 1.0×10^9^/L. Peripheral smear showed marked prominence of plasma cells with occasional binucleate cells. These had abundant basophilic cytoplasm and eccentrically placed nucleus with clumped chromatin [Figure 1]. Many cells showed fine cytoplasmic membrane projections. Bone marrow aspirate and biopsy revealed a near replacement of the normal marrow elements by sheets of plasma cells.

**Figure 2:**
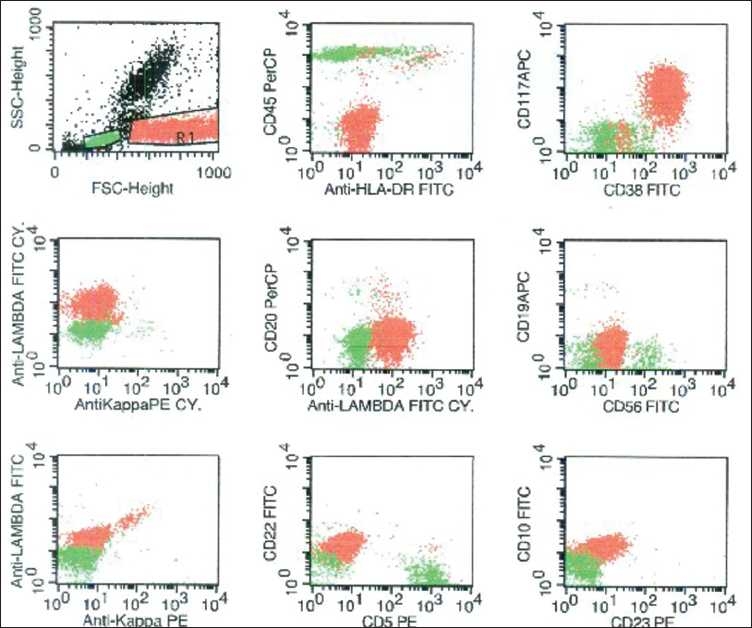
Flow cytometry on the peripheral blood showed the plasma cells (painted red) in the monocytoid region in the forward scatter/side scatter plot. These cells expressed CD38, CD117 and cytoplasmic lambda light chain. These were negative for CD45, HLA-DR, CD20, CD19, CD56, cytoplasmic kappa, surface light chains, CD5, CD22, CD10, and CD23. Normal lymphocytes are painted green

Flow cytometry was performed on the ethylenediaminetetraacetic acid (EDTA)-anticoagulated peripheral blood sample using the lyse-wash method. Surface markers were stained directly and cytoplasmic kappa and lambda chains were stained following the fixation by paraformaldehyde and permeabilization by sapronin. The sample after processing was acquired on FACS Calibur (BD Biosciences, San Jose, CA, USA) and analyzed with the help of CellQuest software. The population with high forward scatter and a slightly high side scatter (corresponding to the monocytoid region) was gated. These cells were negative for CD45 and CD19 and expressed bright surface CD38, CD138, moderate CD117 and showed cytoplasmic lambda light chain restriction [Figure 2]. These cells were negative for CD56, HLA-DR, CD20, cytoplasmic kappa and surface light chains.

Total serum protein was 4.60 g/dL (6–8 g/dL) and albumin was 3.43 g/dL (3–5.2 g/dL). Serum protein electrophoresis did not show an M-band. Serum immunoglobulins quantified by immune-turbidimetric method revealed total IgG 3.31 g/L (7.0–16.0 g/L), IgA 0.09 g/L (0.7–4.0 g/L) and IgM 0.08 g/L (0.4–2.3 g/L). IgE levels evaluated by chemiluminescence immunoassay were 8.6 IU/mL (<100 IU/mL). Free light chains assayed by the turbidometry method showed lambda 3527 mg/L (5.71–26.3 mg/L), kappa 1.15 mg/L (3.3–19.4 mg/L) and a markedly decreased kappa–lambda ratio 0.00033 (0.26–1.65). The serum IgD level was not assessed. Serum calcium was 9.5 mg/dL (8.5–10 mg/dL) and creatinine was 0.6 mg/dL (0.2–1.2 mg/dL). β_2_-microglobulin level in the serum was 12.95 mg/L (<2.19 mg/L) and lactate dehydrogenase (LDH) was 390 U/L (<300 U/L).

**Figure 3:**
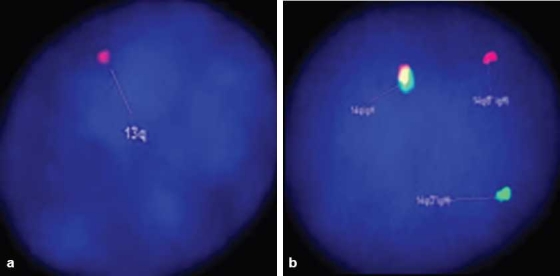
(a) Interphase FISH analysis showing loss of one copy of chromosome 13/13q, indicated by presence of single red signal (arrow). LSI D13S319 DNA probe hybridizes to the band 13q14.3 (red signal) (DAPI counterstain, ×1000). (b) Interphase FISH analysis showing 1red– 1green– 1yellow fusion signal pattern; as compared to the normal cells with a pattern of 0red– 0green– 2yellow (not shown). The splitting of yellow signal into 1red and 1green indicates translocation involving chromosome 14q32 (IgH region) (DAPI counterstain, ×1000)

Karyotyping was performed on the heparinized peripheral blood by directly terminating one part and incubating another part in MarrowMAX (Invitrogen, Carlsbad, CA, USA) medium for 18–20 hours before termination. Using GTG banding technique, 20 metaphases were counted and analyzed, which revealed a normal karyotype (46,XX). Interphase FISH was performed on the heparinized peripheral blood for detecting the following abnormalities: deletion (del)(17p), del(11q), del(13q) and translocation involving chromosome 14q32, i.e., the heavy chain (IgH) region – t(4;14)/t(11;14). The following probes were used respectively: Kreatech p53 (17p13.1) (labeled in orange and green); Kreatech LSI ATM probe (11q22.3); LSI D13S319 (13q14.3) and Kreatech IgH (14q32) break apart probe. 80% of the interphase cells showed del(13q) [Figure 3a] and 16% of the cells showed a translocation related to IgH gene [Figure 3b]. There was no del(17p) or del(11q) detected in these cells.

Radiographs of the skull, dorsal spine, lumbar spine and both femora did not reveal any lytic lesions. The patient was diagnosed as PPCL with aberrant CD117 expression, light chain type and complex translocations involving del 13q and translocation 14q. She thereafter received chemotherapy with the RVd regimen comprising lenalidomide(R), bortezomib(V), and dexamethasone(d) for four cycles at 21 day intervals. After four cycles, she achieved a complete remission (CR), i.e., immunofixation negative CR, absence of plasma cells in the peripheral blood and marrow with 2% plasma cells. Serum free light chain assay revealed normal kappa (8.1 mg/L) and lambda (5.5 mg/L) levels with a normal kappa–lambda ratio (1.47). She underwent high dose chemotherapy with Melphalan 200 mg/m^2^, followed by autologous stem cell transplantation. She continues to be in complete remission, i.e., 9 months since the time of diagnosis.

## DISCUSSION

PPCL is a distinct clinicopathological entity because its presenting features, response to chemotherapy, and prognosis are different from those of typical MM.[5] The development of MM is known to be a result of a multiple step transformation process.[5] The constellation of adverse prognostic factors in patients with advanced aggressive myeloma is already present at diagnosis in patients with PPCL.[5]

Our patient was very young as compared to the usual age of presentation for PPCL, which is 50–60 years.[1–3,5,7] She presented with usual symptoms of PPCL, i.e., features suggestive of anemia and malignancy.[1–3,5,7] These patients have a lower incidence of bone lesions as compared to secondary PCL.[1,2,5,7] In general, PPCL patients have a higher incidence of extranodal presentation, hypercalcemia and renal function impairment; however, our case had none.[1–3,5,7] What could not be explained was the cause for skin rash and itching, whether it was related to the disease or not or was there any skin infiltration.

The immunophenotypic profile is known to be different in MM and PCL.[6,7] Plasma cells usually express CD38 and CD138 both in MM and PCL.[6,7] However, the CD20 displayed a higher reactivity in PCL as compared to MM.[6,7] In addition, the expressions of CD56, CD117, HLA-DR and CD9 were higher in MM than in PCL.[6,7] However, there was no difference noted in the primary and secondary forms of PCL in clinical practice.[6,7] CD28, a research molecule, was known to be expressed more frequently in secondary than in PPCL.[5] The present case expressed CD117, which is uncommon.

A higher proportion of myeloma with light chain only, IgD, or IgE present as PCL as compared to those with IgG or IgA.[6,7] These patients have increased LDH and β_2_-microglobulin serum levels.[5–7] Due to markedly raised β_2_-microglobulin levels, the present case was categorized in stage III according to the International Staging System for Multiple Myeloma, which corroborated with other studies.[6,7]

The molecular abnormalities are known to be commonly occurring in MM and PCL.[6,7] In PPCL, there was a very high incidence of chromosome 13 monosomy, as compared to MM.[1,7] This abnormality was associated with a short survival in MM treated with either conventional chemotherapy or high dose therapy.[7] Amongst the structural abnormalities, studies show a high incidence of chromosome 14q32 translocations with various rearrangements – t(6;14)(p21.1;q32.3), t(11;14)(q13;q32.3), t(14;18)(q32.3;q21.3) and many more.[1,2,8–10] The 14q32 translocations were associated with leukemic transformation and elevated LDH, indicating high myeloma cell mass and advanced disease.[8] Our patient showed both these abnormalities. Other common abnormalities detected in PPCL were chromosome 1 changes, monosomy 7, trisomy 18 and monosomy X in women as compared to trisomies 1, 6, 9, 11, and 15 found commonly in MM.[7] Chromosome 1 changes, both structural and numerical, were frequent in PPCL.[1,2,7–9] However, these were detected in association with 14q32 translocation, indicating secondary karyotypic evolution rather than a primary event.[8] Evaluation of chromosome 1 was not available in our study because of lack of suitable probes.

Presence of abnormal cytogenetic abnormalities in plasma cell dyscrasias is 30–65%.[10,11] The reasons for lack of abnormalities detected on karyotyping could be low proliferation rate of malignant cells, difficulty in obtaining good quality spreads, blurred and contracted chromosomes, resistance of plasma cells to G-banding and possibly the cells examined cytogenetically were not plasma cells.[11] In our case, any one of the above could be the reason for the failure to detect the karyotypic abnormalities, which were detected by FISH, and also an additional factor would have been the use of peripheral blood rather than bone marrow.

As PPCL is a rare malignancy, there is no universal treatment strategy.[5,12] However, due to a dismal prognosis, these need to be treated aggressively as acute leukemias.[12] The principle of treating is achievement of CR with induction chemotherapy followed by stem cell transplant.[12] Various regimens have been tried with a variable degree of success.[3,5,13,14] Recently, the combination of lenalidomide with bortezomib and dexamethasone has shown to be very active in newly diagnosed, refractory and relapsed cases of MM patients, with high response rates and manageable toxicity.[14,15] Although use of this regimen in PPCL has not been documented, we used this combination and the patient attained CR. Autologous stem cell transplantation is the standard transplant option.[12,13] The patient was alive and in CR without any complaints at the time of documentation.

The prognosis for primary and secondary PCL is poor with a median survival of about 6–8 months.[12] The survival is slightly more than 1 year in patients who respond to treatment as compared to less than a month for the non-responders.[5] Although various factors have been implicated in deciding the prognosis, there are many new ones that need to be studied, such as the molecular profile, response to the treatment and the rate at which CR is achieved.[5–7]

PPCL is a rare disorder associated with numerous chromosomal and molecular abnormalities. How these abnormalities contribute to the pathogenesis is yet to be understood. Possibly in future, this understanding would be utilized to develop targeted therapies that would result in better outcome.
